# Correction: Microwave-assisted synthesis of polypyridyl ruthenium(ii) complexes as potential tumor-targeting inhibitors against the migration and invasion of Hela cells through G2/M phase arrest

**DOI:** 10.1039/d0ra90091e

**Published:** 2020-08-26

**Authors:** Jieqiong Cao, Qiong Wu, Wenjie Zheng, Li Li, Wenjie Mei

**Affiliations:** College of Pharmacy, Jinan University Guangzhou China tzhwj@jnu.edu.cn; Integrated Chinese and Western Medicine Postdoctoral Research Station, Jinan University Guangzhou China; School of Pharmacy, Guangdong Pharmaceutical University Guangzhou China wenjiemei@126.com; Department of Chemistry, Jinan University Guangzhou China

## Abstract

Correction for ‘Correction: Microwave-assisted synthesis of polypyridyl ruthenium(ii) complexes as potential tumor-targeting inhibitors against the migration and invasion of Hela cells through G2/M phase arrest’ by Jieqiong Cao *et al.*, *RSC Adv.*, 2017, **7**, 29925, DOI: 10.1039/C7RA90067H.

The authors regret errors in Fig. 1a in the previous versions of the article. The corrected Fig. 1 is shown below, where the panel for the wound healing assay of Hela cells after treatment with **4** (2 μM) has been replaced.

**Fig. 1:**
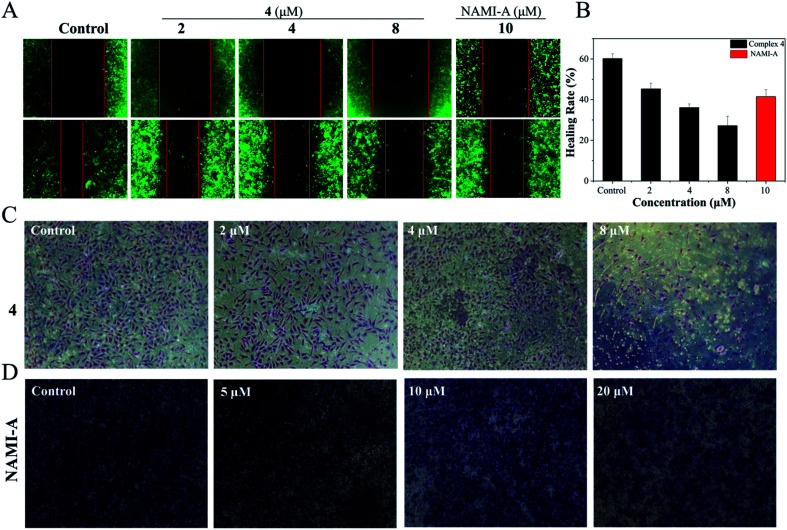
(A) The wound healing assay of Hela cells after treatment with **4** (0, 2, 4 and 8 μM) and [NAMI-A] = 10 μM. (B) The healing rate of Hela cells treated with **4** and NAMI-A. (C) The transwell assay of Hela cells after treatment with **4** (0, 2, 4 and 8 μM) and (D) [NAMI-A] = (0, 5, 10 and 20 μM).

The Royal Society of Chemistry apologises for these errors and any consequent inconvenience to authors and readers.

## Supplementary Material

